# Structural Prediction and Mutational Analysis of Rv3906c Gene of* Mycobacterium tuberculosis* H_37_Rv to Determine Its Essentiality in Survival

**DOI:** 10.1155/2018/6152014

**Published:** 2018-08-15

**Authors:** Md. Amjad Beg, Shivangi, Sonu Chand Thakur, Laxman S. Meena

**Affiliations:** ^1^Centre for Interdisciplinary Research in Basic Science, Jamia Millia Islamia, Jamia Nagar, New Delhi 110025, India; ^2^CSIR-Institute of Genomics and Integrative Biology, Mall Road, Delhi 110007, India

## Abstract

The emergence of tuberculosis is at the peak; therefore to station it at its lower level we hereby try bioinformatics approach against* Mycobacterium tuberculosis* [*M. tuberculosis*] pathogenesis. Rv3906c is a conserved hypothetical gene of* M. tuberculosis* and contains many GTP binding protein motif DXXG which demonstrate that this gene might be processed in a GTP binding or in GTP hydrolyzing manner. This gene shows interaction with its adjacent genes as well as* pcnA *which is a polymerase and localized in the extracellular region and found to be a soluble protein. Rv3906c has binding pockets for calcium atom at various positions which prove that calcium might have some role during the process of this gene. GTP binding protein motif DXXG is present in various positions and calcium binds at this site with a C-score of 0.25. Mutational analysis on this motif shows the large decrease of stability after mutation of aspartate residue with glycine. Stress conditions like pH and temperature also change stability of the protein. A decrease in stability at this position might play a role in inhibition of survival of the pathogen. These computational studies of this gene might be a successful step towards drug development against tuberculosis.

## 1. Introduction

*Mycobacterium tuberculosis* H_37_Rv (*M. tuberculosis*) is a gram-positive and aerobic bacterium [1]. This strain is available all around the environment and enters the host nasal track via inward breath as these pathogens are available in air droplets and discharged through sniffling or hacking of tuberculosis patient [2]. At last, this bacterium reaches to the lungs where it persists for the longer period of time in the alveolar macrophages and becomes successful in causing an active disease [3]. In the alveolar macrophages, it persists for the longer period of time in latent phase, i.e., without showing any symptom of the disease. In the macrophages, it replicates and gets arranged with other immune cells which results in the formation of a granuloma-like structure which is a sign of an active disease [4]. In year 2016, there were 6 million new cases showing resistance to rifampicin (RRTB) [5], the major effective first-line drug, of which 4.9 million had multidrug-resistant TB (MDR-TB) and, among these, almost half (47%) of these cases were in India, China, and Russian countries [6]. The first milestones of the End TB Strategy are set for 2020. In 2017, there were 35% reduction in TB deaths and 20% reduction in TB incidence, compared with levels in 2015. In 2017, WHO (http://www.who.int/tb/publications/global report/en/) has developed TB-Sustainable Development Goals [SDG] monitoring framework of 14 indicators that are covered under seven SDGs associated with TB incidence [7]. Furthermore the worldwide numbers of new and backslide TB cases both have been expanding since 2013 with the noticed rate per 100,000 populace, generally clarified by a 37% expansion only noticed in India [8]. The most imperative factor related to ailment seriousness is the advancement of antimicrobial resistant strains, including multidrug-resistant (MDR) TB and extremely drug-resistant TB (XDR-TB) [9]. TB has an analysis of well-being and symptomatic test; for example, PCR and spreading [10] are exorbitant and tedious regardless that we now require another key to control TB. There are some measures that are not all that affordable like TB biomarker test which is precise and not all that expensive for the TB distinguishing resistant of TB tolerance [11]. For the most part, TB is overwhelming in South Asia, India, and East Africa [12]. Danger of TB event likewise related to kind of organ transplantation and lung transplantation is at high hazard. In present days, Human Immunodeficiency Virus Infection (HIV) and TB tranquillize protection is a worldwide test [13].* M. tuberculosis* and HIV together debilitate the insusceptible framework by diminishing CD4+ T lymphocytes and lessening the host survival [14]. Individuals with this codisease have troublesome treatment result because of official multifaceted nature of these pathogens [15]. Antiretroviral treatment (ART) is prescribed for HIV-TB cotainted people in beginning period of contamination since it indicates expanded HIV-TB cocontaminated host survival [16]. TB has convoluted and dependable impacts on the human body that are not normal but rather might be a risk to life. In 1.5% to 3.5% cases, deep vein thrombosis (DVT) is related to TB and basic to confine TB patients who are at high hazard. Antitubercular treatment (ATT) and anticoagulant treatment can keep the deadly complexity of this illness [17].

So as to comprehend malady pathogenesis, it is important to depict the particular highlights of* M. tuberculosis* that empower it to avoid the host barrier framework and add to its destructiveness [18]. Here, we have explored the characteristics of a small Guanosine triphosphate (GTP) binding protein (G-proteins) of* M. tuberculosis* Rv3906c, which shows so many GTP binding motif in its amino acid sequence. Rv3906c is a conserved hypothetical protein of this bacterium and contains several GTP and calcium binding motifs in its sequence [19]. As GTPases are known to assume a critical part in the survival and pathogenesis of different pathogens, therefore the genes which bind to GTP also play important role in its survival inside the host macrophages which prove Guanosine triphosphatases (GTPases) as subatomic switch proteins [20]. The key part of these proteins includes obstruction in capturing phagosome development, empowering pathogens to get protected from getting away from lysosome and harmful free radicals induced as innate immune responses of the host after infection by this bacterium. This perception gives another road to the advancement of hostile to TB drugs [21, 22]. In a previous couple of years, broad work has been done to comprehend the part of GTPases in the development and advancement of microbes. G-proteins are highly conserved signalling molecules that participate in cellular signalling and bacterial pathogenesis by regulating the activity of cognate GTPases [23–25]. These proteins particularly tie and hydrolyze GTP, which thus endorses or inactivates GTPases in a cyclic way. GTPases are exceptionally monitored and work through RNA or ribosome authoritative. G1, G2, G3, and G4 premise are in charge of particular cooperation with the guanine nucleotide and effectors proteins [26]. The initial two components are associated with communications with the phosphate portion of the GTP atom and the last component is engaged with nucleotide specificity [27]. The consensus sequence contains three consensus sequences GXXXXGK, DXXG, and NKXD [24]. Therefore, in this manuscript, we show various bioinformatics aspects as structural, functional, and mutational studies of gene Rv3906c of* M. tuberculosis* H_37_Rv which might empower experimental work in this field and might be found a suitable antituberculosis drug as shown in Table S1 [28–31].

## 2. Methods and Material

### 2.1. Retrieval of Target Protein Sequence

FASTA proposes grouping of Rv3906c was extricated from Mycobrowser (https://mycobrowser.epfl.ch/genes/) genomic and proteomic database. Rv3906c has 169 amino acid sequence and calcium binding protein homology from* Halobacterium* species. Rv3906c has a GTP binding motif DXXG. There is also the presence of some metal ion binding sites like for calcium and magnesium. Protein database of National Center for Biotechnology Information (NCBI) is an arrangement of alignment from other sources and interpretation from observation code region in value reservoir RefSeq, SwissProt, and Protein Data Bank (PDB). Protein alignment is a noteworthy component of accepted structure and function [32].

### 2.2. SAPS

SAPS (SAP Application Performance Standard) https://www.ebi.ac.uk/Tools/seqstats/saps/ is the statistical analysis tool (using statistics). It works by utilizing the FASTA arranged amino acid groupings and has got the compositional investigation, charge dispersion examination, repetitive structure, multiple's periodicity investigation, dividing inspection [33].

### 2.3. SOSUI Server Tool

SOSUI server was utilized to estimate physicochemical parameters of theoretical proteins. Protein of interest can be submitted in a type of protein arrangement. This server categorizes the protein into cytoplasmic or transmembrane nature. An amphiphilic list of amino acid sequence was produced for enhancing the approach for transmembrane helix prediction. Amphiphilic amino acids have been combined into a framework by using (SOSUI server tool, http://harrier.nagahama-i-bio.ac.jp/sosui/sosui_submit.html) and used for the isolation of coat proteins and the estimation of the transmembrane helical region. Amino acid sequences of soluble proteins and membrane proteins are based on sequence identity cutoff of 25%. Lysine, arginine, tyrosine, and tryptophan amino acids present at the end of region transmembrane helices appear in nature. Amphiphilicity values are positive for polar residue with large hydrophobic stem beyond the *γ* carbon and small polar residue and hydrophobic residue have an amphiphilicity value of zero [34].

### 2.4. STRING Database Server

STRING database server is used for showing the protein-protein interaction between two genes. In the cell cytoplasm, a protein may interact with other proteins and work in the web-like manner. The connections include direct (physical) and indirect (functional) associations; they branch from computational prediction, from sequence convey between organisms, and from interactions aggregated from other principal databases [35]. The number of associations stored in STRING, https://string-db.org/cgi/network.pl?taskId=BUe3enVFzh8M, is shown separately for each data confirming cutoff value ranges between 0 and 1 as low confidence: scores <0.4; medium: 0.4 to 0.7; high: >0.7.

### 2.5. Protein Subcellular Localization Prediction

LocTree3 server is used for Protein Subcellular Localization Server which predicts protein localization sites [36]. LocTree3 is applied on machine learning (profile kernel SVM) to predict the native subcellular localization. The LocTree3 server has a database of 18 classes for eukaryotes. The method outputs a score that reflects the reliability of each prediction. LocTree3 reached an 18-state accuracy of *Q*18 = 80 ± 3% for eukaryotes and a six-state accuracy of *Q*6 = 89 ± 4% for bacteria. LocTree3 (https://rostlab.org/services/loctree2/) server predicts the protein which is present in plasma membrane, nucleus, and membrane bound organelles [37].

### 2.6. Ab Initio Modelling of Protein Sequences

I-TASSER (Iterative Threading Assembly Refinement-https://zhanglab.ccmb.med.umich.edu/I-TASSER/) is used for the protein structure and function prediction. I-TASSER needs the sequence in FASTA format. It builds the 3D model of protein by Ab Initio modelling approach. The server is an online platform for protein structure and function predictions [38]. I-TASSER follows three stages to anticipate the 3D model of the protein. For improving diagram of the secondary structure of a protein by secretly introducing local metathreading server (LOMETS) it uses H, E, and C articulate for alpha-helix, beta-sheet, and coil, respectively. In Rv3906c, optional structure is 2% H, 1% E, and 95% C. The dissolvable directness is isolated into three states by 2 cutoffs esteems: 10% and 40% with the goal that the three states have measured up to distribution, i.e., covered for fewer than 10%, uncovered for bigger than 40%, and medium for the vicinity of 10% and 40%. Covered, medium, and exposed are likewise abridged as B, M, and E, which are 38% E, 42% M, and 19% B; then the component gets simultaneous recreation which is performed by SPICKER bunch centroids and TM-adjust is utilized for LOMET layouts and PDB structure. REMO fabricated the nuclear detail of protein.

In Monte Carlo theory, the confidence of each model is quantitatively measured by the C-score. By the I-TASSER output has ranked top 5 models by the cluster size; possibly lower rank model has a high C-score but the model which is first has a better quality in most cases. C-score is a confidence score for estimating the superiority of the predicted models by I-TASSER. It is typically in the range of -5 to 2, where a higher value signifies a model with a high confidence and vice versa [39, 40].

### 2.7. Prediction of Ligand Binding Pocket

The expectation of the dynamic pocket of our displayed protein was predicted by COACH online server. Before dynamic site-specific molecular docking (binding analysis), the assurance of actual pocket is essential. Binding pocket is the site of the protein where the small ligand can reversibly or irreversibly bind. Just a couple of amino acid deposits are in charge of the ligand. However other amino acids buildups of protein are given accurate introduction and affirmation for the ligand binding site expectation approach for COACH (https://zhanglab.ccmb.med.umich.edu/COACH/) server [41]. COACH server is a metaserver, it begins from given structure of target protein; then it will create correlative ligand binding site prediction utilizing the two relative techniques, TM-site and S-site, which recognize ligand binding arrangement from the database (BioLiP) protein work database by binding particular substructure and collection profile examination. [42]. In the COACH server, output has ranked top 10 model by the cluster size, given C-score, PDB hit, ligand name, complex structure download, and consensus binding residue. Range values of C-score prediction lie between 0 and 1, where the highest score show more reliability. The majority of the proteins (=90%) can be modelled with a correct fold (TM-score>0.5) and 65% have a RMSD below 6Å, although all close homologous templates were excluded in the model generations.

### 2.8. Validation of Modelled Protein Structure

The modelled protein has to be validated and it is done by online server RAMPAGE (Ramachandran Plot Analysis) based on an assessment of Ramachandran Plot. The RAMPAGE server approves the protein structure on the premise of *φ*, *ψ* point of individual deposits [43, 44]. The validation of protein was performed by the structure analysis and verification server version 4 (SAVES) is a metaserver which checks the stereochemical quality of a protein structure by analyzing residue-by-residue geometry and overall structure geometry [45–47]. This metaserver runs 6 programs for checking and validating protein structures during and after model refinement. In our model refinement we use Ramachandran Plot (http://services.mbi.ucla.edu/SAVES/Ramachandran/) [45], ERRAT (http://services.mbi.ucla.edu/ERRAT/) [46], Verify 3D (http://services.mbi.ucla.edu/Verify_3D/) [47, 48], and PROVE [49].

### 2.9. Structure-Based Function Prediction

Structure-based function prediction is predicted by the COFACTOR (https://zhanglab.ccmb.med.umich.edu/COFACTOR/) online server. It is a structure, arrangement, and protein-protein collaboration based procedure for an organic explanation of protein atoms [50]. Beginning from the 3D basic model, cofactor will run the problem through the BioLiP protein function database by nearby and universal structure matches to distinguish functional conditions and homologies. Practical bits of knowledge, including gene ontology (GO), Enzyme Commission (EC), and ligand restricting locales, will get through the best practical homology layouts. For GO, which means gene ontology, extra knowledge will be acquired from Gene Ontology Annotation (UniProt-GOA) UniProt-GOA by succession and grouping profile arrangements and from STRING by protein-protein collaboration inferrals. The COFACTOR structure-based function prediction calculation was positioned as the best strategy for protein work forecast. We applied this approach for the prediction of molecular function and biological process. In the COFACTOR server, Cscore^GO^ is the confidence score of predicted GO terms. Cscore^GO^ values range within [0-1], where higher value indicates a better confidence in predicting the function using the template [51, 52].

### 2.10. Mutational Analysis

In proteomics and genomics, contemplating studies of protein strength, free energy change (ΔΔG) upon single point mutation may likewise enable the explanation of process. The test ΔΔG esteems are influenced by susceptibility as measured by standard deviations. The greater part of the ΔΔG esteems are almost zero (around 32% of the ΔΔG informational index ranges from −0.5 to 0.5 kcal/mole) and both the esteem and indication of ΔΔG might be either positive or negative for a similar change obscuring the relationship among mutated and expected ΔΔG esteem. Keeping in mind the final goal to conquer this issue, we portray another indicator that segregates between 3 change classes: destabilizing mutations (ΔΔG<−1.0 kcal/mol), balancing out transformations (ΔΔG>1.0 kcal/mole), and nonpartisan changes (−1.0≤ΔΔG≤1.0 kcal/mole). For the I-MUTANT 3.0 suite calculation, DDG<-0.5 means (large decrease of stability), DDG>0.5 means (increase if stability), and -0.5<=DDG<=0.5 means (neutral stability) [53–55]. For the prediction of protein, stability change upon a single point mutation was predicted by I-MUTANT 3.0 Suite server (http://gpcr2.biocomp.unibo.it/cgi/predictors/I-Mutant3.0/I-Mutant3.0.cgi).

## 3. Results

### 3.1. Retrieval of Target Protein Sequence

FASTA format nucleotide, as well as protein sequence of Rv3906c, has been retrieved from the Mycobrowser database. Rv3906c is 510 bp long gene and 17 kDa protein. It is associated with cell wall and its adjacent functions. It is a conserved hypothetical protein and not studied before.

### 3.2. SAPS

According to saps result, we found that Rv3906c is glycine and aspartate-rich protein. According to the statistical analysis of SAPS, this gene contains 17.8% glycine and 23.7% aspartate.

### 3.3. SOSUI Server Tool

A SOSUI server tool result shows that Rv3906c is not a transmembrane protein. It is a soluble protein with normal hydrophobicity of -0.453846.

### 3.4. Protein-Protein Interaction

STRING database server result shows that Rv3906c interacts with Rv3902c, Rv3903c, Rv3904c, Rv3905c, and* pcnA* (Rv3906c), which is poly A polymerase gene. According to string database server, Rv3906c interaction result with other proteins has minimum interaction score [between 0.4 and 0.6] shown in Table 1, but it shows high interaction score for* pcnA,* i.e., 0.785.

### 3.5. Protein Subcellular Localization Prediction

**Figure 1:**
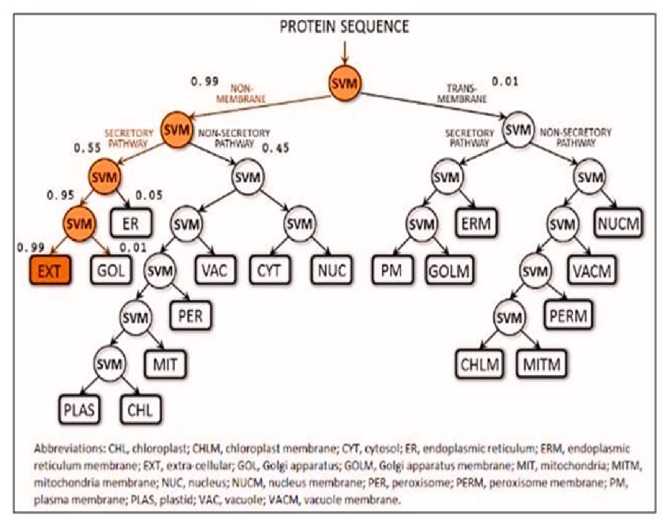
**Prediction of localization by LocTree3**. LocTree3 tool used for determining protein subcellular localization which shows that it is an extracellular regional, secreted protein. The figure shows that 0.99% of this protein is a secretory protein which is present in extracellular region. 0.01% of this protein is present in transmembrane region.

According to LocTree3 server, Rv3906c is a secreted protein, present in the extracellular region with the accuracy of 89% which is shown in Figure 1.

### 3.6. Structure Prediction of Rv3906c via I-TASSER

**Figure 2:**
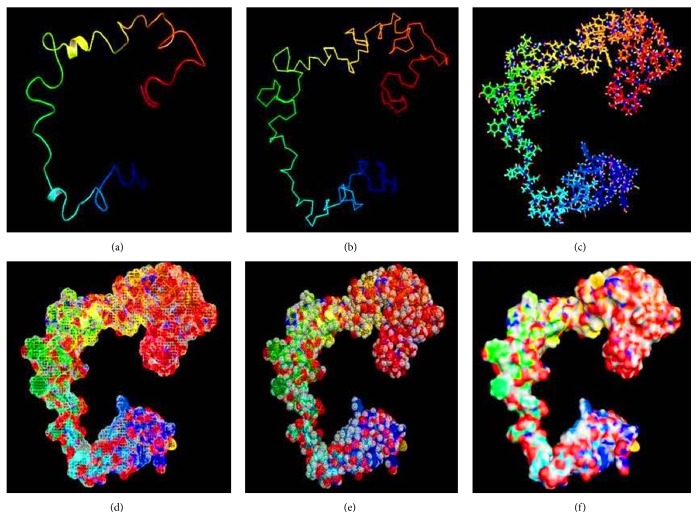
**Modelling of protein via I-TASSER**. Rv3906c protein modelled via I-TASSER showing C-score -1.37, estimated RMSD 0.55±0.15, and estimated TM-score 7.9±4.4Å. (a) Cartoon model, (b) ribbon model, (c) sticks model, (d) mesh model, and (e) dot model of (f) surface model.

The structure of the Rv3906c protein was modelled by I-TASSER. The quality of modelled protein depends upon the percentage of the favorable region which lies above 90% of the value of C-score and RMSD value. The C-score is the confidence score for each model. It is computed by threading layouts arrangement. The C-score changes inside the range from -5 to 2 and higher certainty is controlled by the higher estimation of C-score. Finally, I-TASSER creates top 5 models according to C-score, positioned by group measure among which the figure with higher C-score is shown in Figure 2.

### 3.7. Prediction of Ligand Binding Pocket

**Figure 3:**
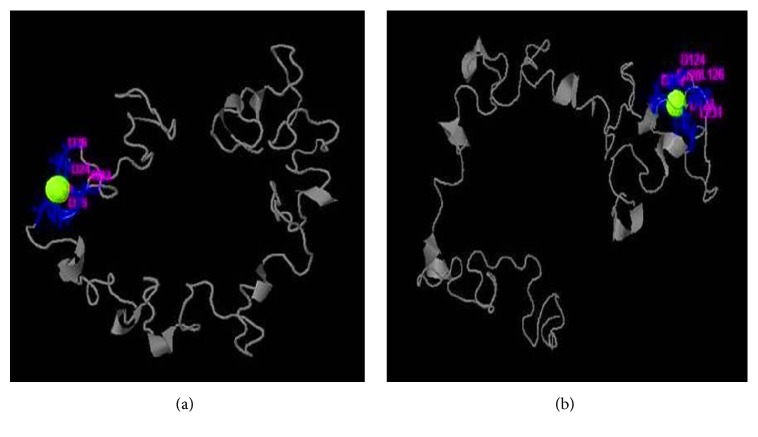
**Prediction of calcium binding pocket in modelled protein by COACH server**. The figure shows calcium ligand binding in pocket of Rv3906c protein. (a) Calcium binds to aspartate residue at 22, 24, 26, 33, and 35 positions with C-score of 0.25 (rank 1). (b) Calcium binds to aspartate residue at positions 122, 124, 126, and 131 with C-score of 0.23 (rank 2).

Rv3906c has ligand binding site for calcium which is confirmed by coach server. Coach server results show that calcium binding with this gene at rank 1 position has C-score of 0.25. Calcium binds with Rv3906c aspartate residue at 22, 24, 26, 33, and 35 positions. Rank 2 site binds calcium at positions 122, 124, 126, and 131 with C-score 0.23 and including COFACTOR result shows C-score is 0.21, TM-score is 0.776, and RMSD is 2.83. Rv3906c shows so many GTP binding motif DXXG that are important for GTP binding and hydrolyzing activity. Aspartate in this position plays a crucial role for its activity and in this gene aspartate is present in D22, D24, D35, D120, D122, D143, and D145 positions which substantially binds to calcium as shown in Figure 3.

### 3.8. Validation of Protein 3D Structure

After modelling of structure, the protein structure was validated through and SAVES server (RAMPAGE (Ramachandran Plot Analysis), ERRAT, PROVE, and Verify3D).

#### 3.8.1. RAMPAGE Analysis

**Figure 4:**
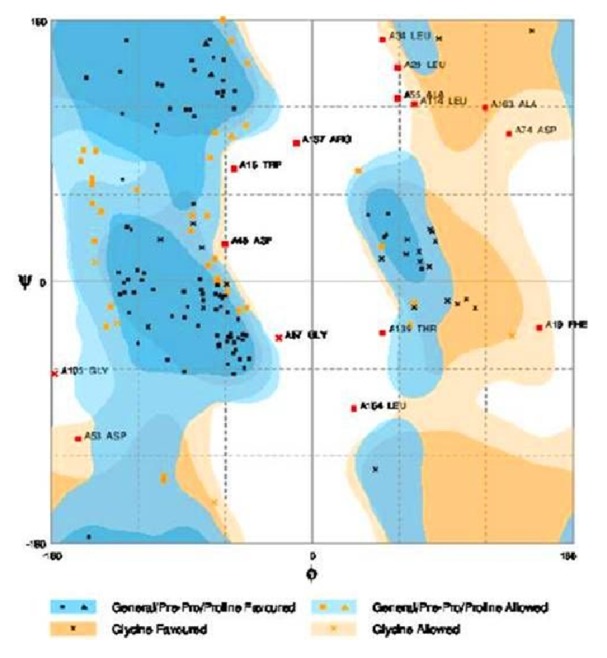
**RAMPAGE analysis of modelled protein**. RAMPAGE analysis shows that in favored region there were 67.7% of residues and in allowed region 23.4% of residues and outlier region 9.0% of residues were present.

The demonstrated protein was validated by RAMPAGE (Ramachandran Plot Investigation) which is an online server. After examination of Ramachandran Plot of our demonstrated protein, the structure demonstrated that 67.7% of residues have been present in a favored region, although other 23.4% of residues were laid in the permitted area and 9.0% of deposits were laid in exception conditions. These parameters of protein structure demonstrate that our displayed protein was of good quality and stable and adequate (Figure 4).

#### 3.8.2. ERRAT

**Figure 5:**
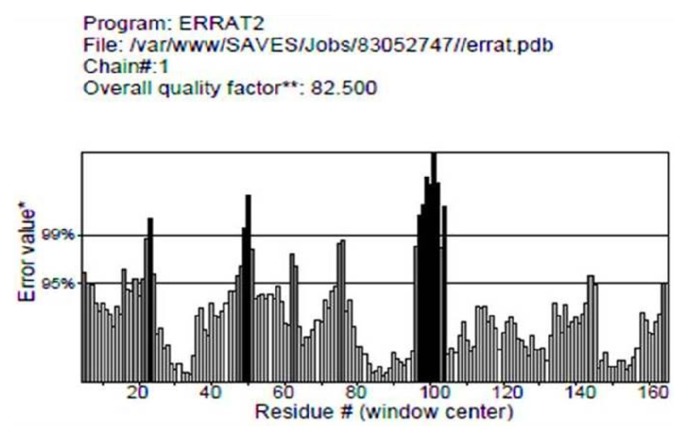
**Structure validation with ERRAT tool**. The result of ERRAT tool shows that overall quality factor of the modelled protein based on various sorts of atoms were found to be 82.500 which are satisfactory.

ERRAT is an online server which approves the protein structure on the premise of the nuclear connection between various sorts of atoms. The overall quality factor is 82.500 of our protein structure shown in Figure 5 which is acceptable.

#### 3.8.3. PROVE [Protein Volume Evaluation]

**Figure 6:**
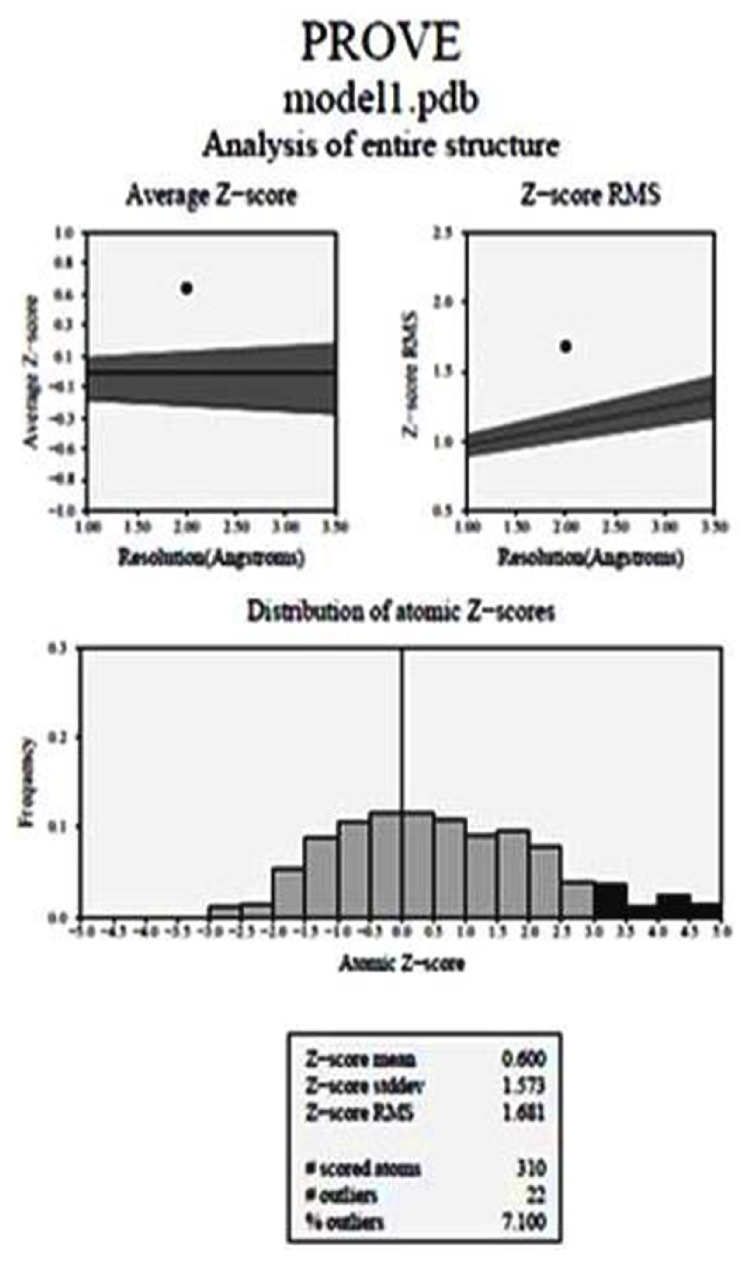
**PROVE analysis of protein**. Z-score defined the energy separation between native fold and average of an ensemble of misfold unit. The average Z-score was 0.600 and the Z-score RMS was 1.681.

The Z-score of a protein is characterized as the energy partition between the local cover and the normal of an outfit of misfolds in the units of the standard deviation of the group. The Z-score is used as a technique for testing the data based conceivable outcomes for their ability to see the nearby overlay. PROVE is an online server that affirmed our entire structure of the shown protein. Z-score is calculated, representing the quantity of standard deviation away from the mean volume of an atom having a similar group. A negative Z-score implies that the atom has a smaller than normal volume, though a positive score shows that a particle has a bigger than normal volume. The normal Z-score is zero. The Z-score RMS deviation from ideality is utilized as a worldwide measure of leaving from the normal conduct in a given arrangement of N atoms, which can be all the atoms of a given protein structure or atom. Figure is demonstrated by the normal Z-score and Z-score RMS values. The normal Z-score of our protein was 0.600 and Z-score RMS was 1.681 which is shown in Figure 6.

#### 3.8.4. Verify3D

**Figure 7:**
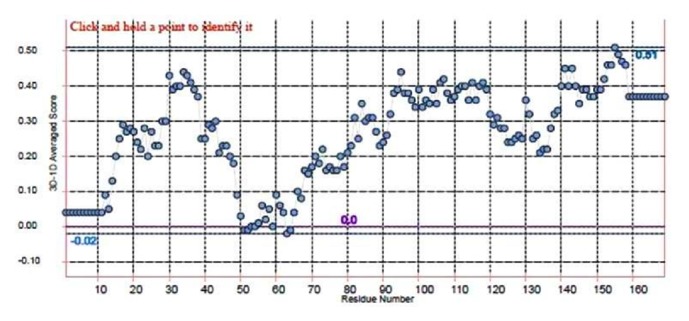
**Analysis of 3D-1D score of modelled protein by Verify 3D**. The figure shows that 74.56% of the residues had an average of 3D-1D score >=0.2 that is acceptable for our demonstrated protein.

The Verify3D strategy evaluates protein structure by utilizing three-dimensional profiles. This program examines the similarity of a nuclear model (3D) with its own particular amino acid sequence which is 1-dimensional. Every deposit doled out a basic class in radiance of its area and condition (alpha, beta, circle, polar, nonpolar, and so on). The score ranges from -1 (poor score) to +1 (good score). 74.56% of the sequence was found in the middle value of 3D-1D score >=0.2 that is perceptive for our demonstrated protein shown in Figure 7.

### 3.9. Structure-Based Function Prediction

**Figure 8:**
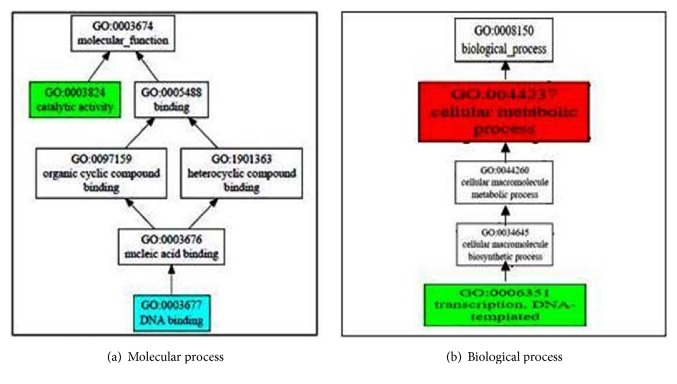
**Structure-based function prediction by cofactor server**. The figure shows gene ontology term with molecular function and biological process. The whole procedure demonstrates the C-score of 0.99 with the cell metabolic process and 0.63 with translation, DNA templated. COFACTOR online server predicts templated protein with comparable binding site which comes at positions 22 and 24 additionally.

The structure-based function prediction is predicted by the COFACTOR online server. It is a structure, arrangement, and protein-protein association based strategy for natural observation of protein particles. COFACTOR results predicted structural analog in PDB, molecular capacity, biological process, cellular segment, enzyme homolog in PDB, and layout protein with comparative binding sites shown in Figure 8. Cscore^GO^ is the confidence score of predicted GO terms. COFACTOR online server results in the anticipated quality metaphysics GO (gene ontology) terms which are arranged by atomic capacity, organic process, and cell part with a definite C-score. In the expectation of quality values in the atomic competence, C-score is 0.68 with synergist action and 0.54 with respected DNA. In the whole procedure, C-score is 0.99 with the cell metabolic process and 0.63 with translation, DNA templated. COFACTOR online server predicts templated protein with comparable binding site which comes at positions 22 and 24 additionally which is exhibited there.

### 3.10. Mutational Analysis

Testability of the protein investigation was predicted by utilizing I-MUTANT 3.0 Suite server. Rv3906c which is theoretical protein has a DXXG motif. From the COACH server we anticipate the ligand binding site at these motif destinations present on aspartate of 22, 24, 35, 120, 122, 143, and 145 position. Moreover, aspartate is found to be essential at this site; therefore, to check the essentiality of this aspartate residue in whole GTP binding and hydrolyzing activity we have to change this residue by all other amino acid residues at particular position.

#### 3.10.1. Change of Aspartate with Other Amino Acid

**Figure 9:**
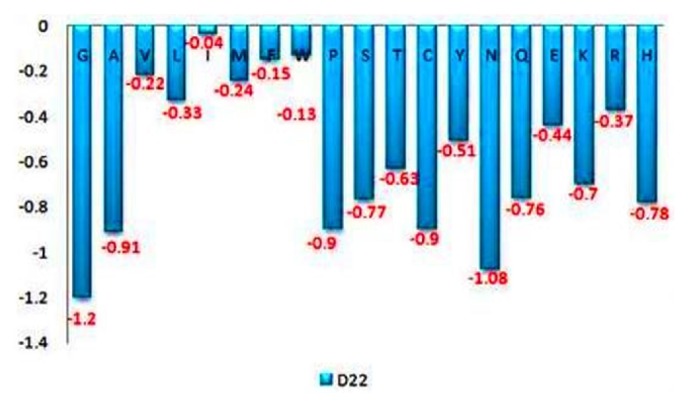
**Prediction of stability change of protein by I-Mutant 3.0 suite**. I-Mutant 3.0 suite predicts stability change upon single point mutation at Aspartate [D22] residue which is important residue for GTP binding activity. The figure emphasizes that changing of aspartate with glycine decreases the stability most with a DDG value of -1.2.

As aspartate is crucial for GTP binding and hydrolyzing activity; therefore mutation at this position with all other amino acid had been shown in Figure 9. In the graph, we have seen that there are more than 12 amino acids that have largely decreased the stability means -0.5 or below value towards negative. So, we select glycine because it has -1.20 DDG values which mean large decrease in protein stability at 37°C temperature and at pH 7.0.

#### 3.10.2. Change in the Position of Aspartate

**Figure 10:**
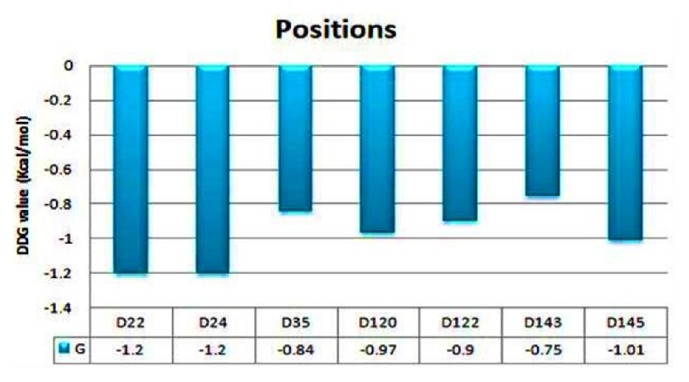
**Difference in stability of the protein by changing position**. The figure shows that aspartate of positions 22 and 24 is the most important site for GTP binding and hydrolyzing activity with a DDG value of -1.2.

At that point, we check the expectation of mutational analysis on aspartate of positions 22, 24, 35, 120, 122, 143, and 145. Thus, we ascertained the destability (implies protein structure is not steady with this change of an amino acid) of the protein on that position by checking the DDG esteem (Kcal/mol). The outcomes appeared in Figure 10. Here mutant type amino acid glycine on the positions 22 and 24 has a similar DDG value of -1.20 when we change aspartate into glycine as shown in Figure 10.

#### 3.10.3. Comparative Studies by iStable Server

In a protein, a single amino acid change can cause mutation which may cause loss of protein function. In the protein stability prediction, the protein stabilizing direction is more significant than knowing its magnitude. iStable (integrated predictor for protein stability change upon single mutation) tool works by using sequence information and prediction results from different element predictors. In the construction of iStable, web-based prediction five chosen element predictor tools were: I-Mutant2.0, PoPMuSiC2.0, AUTO-MUTE, CUPSAT, and MUPRO. For comparative study of determining change in protein stability by iStable server which adopted the support vector machine as an integrator, this server employs two different types of input described as structural and sequential. In the sequential input analysis there are only two predictors used: iStable I-Mutant2.0 and MUPRO.

I-Mutant2.0 and MUPRO adopt a SVM model to approximate the ddG value of the protein and predict the direction of stability change. iStable result does not provide ddG value; it only predicts the stability of the protein either increases or decreases with confidence score. In I-Mutant2.0 server the positive values of ddG show more stabilizing data whereas negative values depict destabilizing data. The comparative study result of mutational analysis by iStable server is shown in Table 2.

#### 3.10.4. Change in Stress Condition

**Figure 11:**
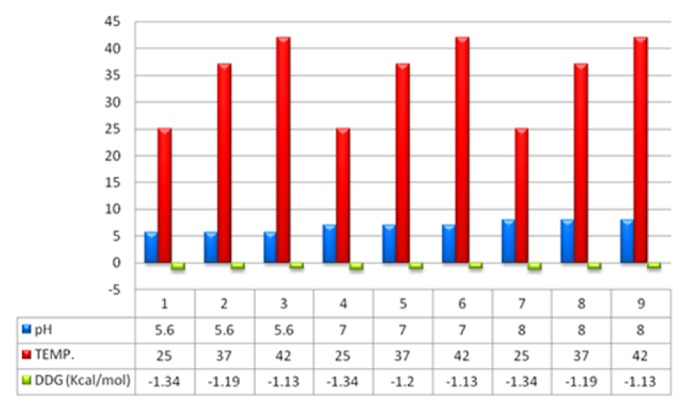
**Change in stability in stress condition**. The figure shows change in stability in stress condition, i.e., pH and temperature. The blue color in figure shows three different pH, i.e., 5.6, 7, and 8 and three different temperatures, i.e., 25°C, 37°C, and 42°C. The graph demonstrates that pH does not affect the stability of the protein whereas by increasing temperature stability decreases by 21%.

After considering the graph result we select an arbitrary position that is aspartate 22 for checking the change in DDG in stress condition. We select three different pH 5.6, 7, and 8 and three different temperatures 25°C, 37°C, and 42°C as stress conditions. Our result from bioinformatics study emphasizes that increasing or decreasing pH does not affect the stability of the protein at all three temperatures whereas, by decreasing the temperature, there is a decrease in the stability of the protein which is shown in Figure 11.

## 4. Discussion

In the present situation, we can see that there is no protective and curative therapy to eradicate tuberculosis completely except BCG. Past decades of research already show that BCG provides limited protection against tuberculosis but fails in protecting MDR, TDR, and XDR cases of tuberculosis. Continuous effort had been put by scientists in order to increase the effectiveness of the vaccine and in searching for new drug targets. In this manuscript, we emphasize Rv3906c gene of* M. tuberculosis* H_37_Rv. After computational analysis of this gene, we find that this gene is 17 kDa proteins by Mycobrowser database [32]. Statistical analysis of this gene by SAPS server [33] confirmed that this protein is rich in glycine-aspartate residues. This protein is a soluble protein which is confirmed by SOSUI server [34] and interacts with its adjacent genes such as* pcnA* which is a poly A polymerase with score 0.785 shown by STRING database [35]. This protein has an accuracy of 89% localization particularly in extracellular region which is confirmed by LocTree3 server [36, 37] as shown in Figure 1. Model prediction of this gene is formed by I-Tasser [38] and validation of the model was done by SAVES metaserver which confirms that the model of the protein has satisfactory output. This gene has many GTP binding motif DXXG which is essential for GTP binding and hydrolyzing activity. Rv3906c contains this motif at residues 22, 24, 26, 33, and 35 positions as rank 1 with C-score of 0.25 and rank 2 at positions 122, 124, 126, and 131 with C-score 0.23 [C-score cutoff is 0-1]. Aspartate in DXXG motif is essential for its crucial activity [19]. For this protein structure we find out the ligand binding pocket by COACH server [41] whose results prove that aspartate in this position has the ability to bind with calcium (Ca) ligand as rank 1 position with C-score of 0.25 and rank 2 with C-score 0.23 and including server studies shows TM-score is 0.776 and RMSD is 2.83 which seems to be satisfactory. RAMPAGE analysis [46] shows that 67.7% of the structure appears to be in the favored region. As we discussed the structure-based function prediction by COFACTOR server [50] whose results show molecular function predicted as catalytic activity with C-score of 0.68 and biological process such as cellular metabolic process with C-score of 0.99. For the analysis of the mutational studies by I-Mutant 3.0 server [54] and iStable server [56] which need an input in form of protein sequence (not in FASTA format) which confirms that the wild type aspartate was mutated with all other amino acids, we have seen that interchange by glycine decreases the stability at maximum by DDG of -1.20. Mutational analysis results in stress conditions showing that decreasing or increasing pH does not affect the stability of the protein structure at all different temperatures whereas a decrease in temperature from 37°C temperature decreases stability (DDG value of -1.34) as shown in Figure 11. After analysis of Rv3906c, we proposed that this gene might possess GTP binding, hydrolyzing, and calcium binding properties that seem to be essential for the vitality of* M. tuberculosis* inside the phagosome of the host. Mutational analysis shows that this gene loses its stability in stress conditions; therefore further experimental studies on this gene might be beneficial to prove this gene as a new drug target.

## 5. Summary

The emergency of tuberculosis is at that level at which we need a safe and secure way to prevent our future generation from this hazard. This strain* M. tuberculosis* is now changing into multidrug-resistant (MDR), extensively drug-resistant (XDR), and totally drug-resistant (TDR) which remain as one of the major challenges. We would ensure this from our attempt to invent a new vaccine or antituberculosis drug. Rv3906c is a hypothetical protein and not studied before. As this gene contains many GTP binding motifs, therefore it can be predicted as a GTP binding and GTP hydrolysis property. As GTP is the very important molecule in eukaryotes as well as prokaryotes, therefore targeting this gene for disruption of its functional characteristics might be an initiative in vaccine development against tuberculosis.

## Figures and Tables

**Table 1:** STRING server protein-protein interactions with Rv3906c protein where score shown separately for each data confirms cutoff value within [0-1], low confidence: scores <0.4; medium: 0.4 to 0.7; high: >0.7.

**S. No.**	**Predicted Functional Partners**	**Predicted function**	**Score**
1	*pcnA* (480aa)	Poly (A) Polymerase	0.785
2	Rv3902c (103aa)	Hypothetical protein	0.653
3	Rv3903c (90aa)	Hypothetical protein	0.639
4	Rv3904c (846aa)	Hypothetical protein	0.639
5	Rv3905c (176aa)	Hypothetical protein	0.639

**Table 2:** Comparative studies by iStable (metaserver) for protein stability prediction confirm that the wild type aspartate on position 22 mutate with glycine (D22G) has the highest decrease in stability.

		**Protein Stability**				
**S. No.**	**Mutation**	**i -Mutant 2.0 server**	**MUpro server**		**iStable server**	
		**ddG value**	**Prediction stability**	**Confidence score**	**Prediction stability**	**Confidence score**
1	D22G	-1.28	Decrease	-1	Decrease	0.805849
2	D24G	-1.28	Decrease	-0.0645742	Decrease	0.786639
3	D35G	-0.84	Decrease	-0.2010039	Decrease	0.703789
4	D120G	-1.01	Decrease	-0.4610863	Decrease	0.728639
5	D122G	-0.87	Increase	0.2685563	Increase	0.65624
6	D143G	-0.89	Decrease	-0.2347231	Decrease	0.604242
7	D145G	-1.17	Increase	0.26545563	Increase	0.648854

## Data Availability

The data used to support the findings of this study are available from the corresponding author upon request.

## Abbreviations

TB: Tuberculosis
*M. tuberculosis*: * Mycobacterium tuberculosis*
AMs: Alveolar macrophages
RRTB: Resistance to Rifampicin Tuberculosis
MDR-TB: Multidrug-resistant TB
SDG: Sustainable Development Goals
XDR-TB: Extremely drug-resistant TB
GTPases: Guanosine triphosphatases
G-proteins: GTP binding proteins
PDB: Protein Data Bank
NCBI: National Center for Biotechnology Information
SAPS: SAP Application Performance Standard
I-TASSER: Iterative Threading Assembly Refinement
LOMETS: Local metathreading server
RAMPAGE: Ramachandran Plot Analysis
UniProt-GOA: Gene ontology annotation.

## Supplementary Materials

Supplementary Materials Table shows the list of different servers used in the study.
