# Supplementary material for: Structural Prediction and Mutational Analysis of Rv3906c Gene of Mycobacterium tuberculosis H37Rv to Determine Its Essentiality in Survival
Source: Adv Bioinformatics. 2018 Aug 15;2018:6152014. doi: 10.1155/2018/6152014 (PMC6114228; doi:10.1155/2018/6152014)
Supplement: Supplementary Materials — Table shows the list of different servers used in the study. [file 6152014.f1.doc]

**Table S1: Supplementary Table**

| **S.No.** | **Tools** | **URL** | **Function** | **Reference** |
| --- | --- | --- | --- | --- |
| 1 | Mycobrowser | https://mycobrowser.epfl.ch/genes/ | For the Sequence retrieval of protein interest and Physio-chemical properties. | 33 |
| 2 | SAPS | <https://www.ebi.ac.uk/Tools/seqstats/saps/> | For the Statistical analysis of the protein. | 34 |
| 3 | SOSUI | <http://harrier.nagahama-i-bio.ac.jp/sosui/sosui_submit.html> | For the prediction of membrane protein or transmembrane protein. | 35 |
| 4 | STRING | <https://string-db.org/cgi/network.pl?taskId=BUe3enVFzh8M> | For the study of protein-protein interaction. | 36 |
| 5 | Loc Tree3 | <https://rostlab.org/services/loctree2/> | For the localization of protein. | 37, 38 |
| 6 | I-TASSER | http://zhang.bioinformatics.ku.edu/I-TASSER | 3D Modelling of protein. | 39-41 |
| 7 | COACH | <https://zhanglab.ccmb.med.umich.edu/COACH/> | For the protein ligand binding site prediction. | 42, 43 |
| 8 | SAVES | https://services.mbi.ucla.edu/SAVES/ | For the protein structure validation. | 44-50 |
| 9 | COFACTOR | https://zhanglab.ccmb.med.umich.edu/COFACTOR/ | For the structure based function prediction. | 51-53 |
| 10 | I-Mutant 3.0 | http://gpcr2.biocomp.unibo.it/cgi/predictors/I-Mutant3.0/I-Mutant3.0.cgi | For the prediction of protein stability change upon a single point mutation. | 54-56 |

**Table S1:** Table showing the list of different servers used in the study
